# A Conceptual Framework to Study the Implementation of Clinical Decision Support Systems (BEAR): Literature Review and Concept Mapping

**DOI:** 10.2196/18388

**Published:** 2020-08-06

**Authors:** Jhon Camacho, Manuela Zanoletti-Mannello, Zach Landis-Lewis, Sandra L Kane-Gill, Richard D Boyce

**Affiliations:** 1 Department of Biomedical Informatics School of Medicine University of Pittsburgh Pittsburgh, PA United States; 2 I&E Meaningful Research Bogotá Colombia; 3 School of Medicine Pontificia Universidad Javeriana Bogotá Colombia; 4 Department of Learning Health Sciences University of Michigan Ann Arbor, MI United States; 5 Department of Pharmacy and Therapeutics School of Pharmacy University of Pittsburgh Pittsburgh, PA United States

**Keywords:** clinical decision support system, computerized decision support system, implementation science, technology acceptance, barriers, facilitators, determinants, decision support system

## Abstract

**Background:**

The implementation of clinical decision support systems (CDSSs) as an intervention to foster clinical practice change is affected by many factors. Key factors include those associated with behavioral change and those associated with technology acceptance. However, the literature regarding these subjects is fragmented and originates from two traditionally separate disciplines: implementation science and technology acceptance.

**Objective:**

Our objective is to propose an integrated framework that bridges the gap between the behavioral change and technology acceptance aspects of the implementation of CDSSs.

**Methods:**

We employed an iterative process to map constructs from four contributing frameworks—the Theoretical Domains Framework (TDF); the Consolidated Framework for Implementation Research (CFIR); the Human, Organization, and Technology-fit framework (HOT-fit); and the Unified Theory of Acceptance and Use of Technology (UTAUT)—and the findings of 10 literature reviews, identified through a systematic review of reviews approach.

**Results:**

The resulting framework comprises 22 domains: agreement with the decision algorithm; attitudes; behavioral regulation; beliefs about capabilities; beliefs about consequences; contingencies; demographic characteristics; effort expectancy; emotions; environmental context and resources; goals; intentions; intervention characteristics; knowledge; memory, attention, and decision processes; patient–health professional relationship; patient’s preferences; performance expectancy; role and identity; skills, ability, and competence; social influences; and system quality. We demonstrate the use of the framework providing examples from two research projects.

**Conclusions:**

We proposed BEAR (BEhavior and Acceptance fRamework), an integrated framework that bridges the gap between behavioral change and technology acceptance, thereby widening the view established by current models.

## Introduction

Every year, significant amounts of resources are invested in medical research globally, an average of 0.19% of the gross domestic product in high-income countries [1]. All this effort has resulted in the exponential growth of scientific evidence. However, the translation of that knowledge into changes in clinical practice is advancing at a much lower rate, creating a growing knowledge-practice gap [2]. Reducing this gap requires not only the development and dissemination of evidence-based guidelines but also the integration of guideline recommendations into care processes and that health professionals change their practice. Clinical decision support systems (CDSSs) present a promising approach to address these challenges [3-6].

CDSSs encode clinical knowledge into computerized algorithms and combine them with patient-specific data to provide clinicians with information and decision guidance [7]. When successfully implemented, the ability of a CDSS to provide patient-specific decision support empowers health professionals to make timely decisions at the point of care while reducing medical errors [8,9]. Another benefit of this technology is that the transformation of clinical knowledge into algorithms allows for the correction of areas where documents (eg, clinical practice guidelines) are ambiguous or unclear [10-13]. CDSSs have been implemented to support care in several specialties [14-20], both in developed and developing countries [14,18,21].

Although several literature reviews have shown improvements in process measures after the implementation of CDSSs [8,22-24], the evidence of their effectiveness on clinical outcomes is still mixed [22,25,26]. This is partially explained because successful implementation of practice-change interventions is a multidimensional problem requiring attention to many factors [27-30]. Relevant factors include not only those internal and external to the health organization [29] but also those related to the clinicians' preferences and their mental model about their practice [28,30]. Additionally, CDSSs must be integrated into the clinical workflow and be accepted by the users.

We hypothesize that improving the implementation of CDSSs requires attention to factors related to practice change and also to those associated with technology acceptance, defined as the user’s decision to use a technology system routinely [31]. Theories and frameworks about these topics can be found in the research fields of implementation science and technology acceptance. However, though drawing from similar sources (ie, psychology, sociology, and management science), these fields have developed into separate disciplines. Therefore, a researcher considering studying the implementation of a CDSS as a strategy to foster clinical change is confronted with a fragmented corpus of knowledge and the choice among conceptual frameworks, potentially missing or having to give up the contributions from one of these fields.

Another issue is that there are competing frameworks within the fields of implementation science and technology acceptance. Several authors have proposed theoretical models to explain the determinants of clinical practice change [32]. Similarly, several models attempt to explain the factors influencing the user’s decision to use a technology system routinely [33,34]. Furthermore, several recent studies have identified determinants that are specific to the acceptance of CDSSs [35-40]. When seen together, these frameworks amount to too many concepts for a reasonable research project to use effectively.

To address these issues, we propose BEAR (BEhavior and Acceptance fRamework), an integrated conceptual framework that bridges the gap between behavioral change and technology acceptance aspects of the implementation of CDSSs. BEAR synthesizes literature about factors influencing both practice change and acceptance of CDSSs from the health professional's perspective. Furthermore, BEAR seeks to capture the variability in the phenomenon of implementing CDSSs while providing an integrated tool that facilitates the design of research and evaluation projects.

## Methods

### Overview

**Figure 1:**
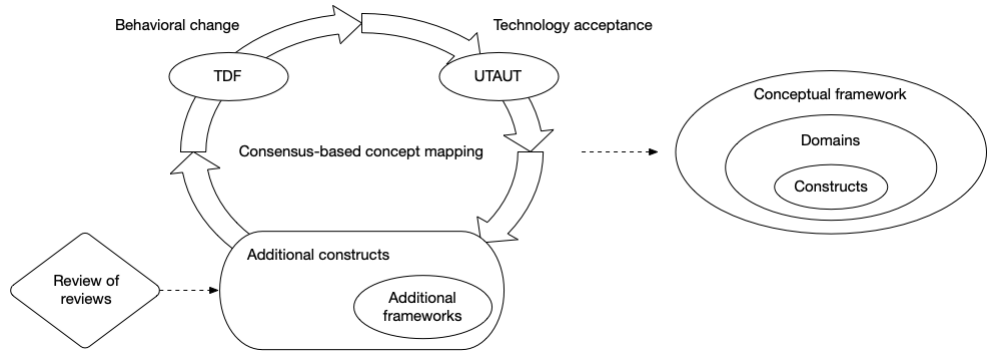
Framework development. TDF: Theoretical Domains Framework; UTAUT: Unified Theory of Acceptance and Use of Technology.

We developed BEAR by employing an iterative process in which two investigators (JC and MZM) mapped constructs reported in the literature as determinants of behavioral change and acceptance of CDSSs (see Figure 1). In each iteration, both investigators mapped the constructs from a framework or a literature review into the emerging construct pool, starting with the Theoretical Domains Framework (TDF) [28,30] and the Unified Theory of Acceptance and Use of Technology (UTAUT) [34]. At the beginning of each iteration, the two investigators (JC and MZM) developed maps independently contrasting the information in the articles with the definitions in the construct pool. After that, the investigators discussed differences in their maps and agreed on modifications to the pool. These modifications encompassed the following: the inclusion of new constructs, in addition to agreeing to their definitions; changes in construct labels or definitions; and changes in the grouping of constructs into domains. The emerging framework was progressively documented in two files: one contained the definitions and the other contained the map to the original constructs (see Multimedia Appendices 1 and 2).

### Base Frameworks

The first iteration comprised the mapping of two well-established frameworks: the TDF [28,30] and the UTAUT [34]. The TDF, proposed by Michie et al in 2005 [30] and revised by Cane et al in 2012 [28], comprises 84 theoretical constructs included in classic psychological theories about behavior change. The UTAUT, on the other hand, was proposed by Venkatesh et al in 2003 [34] to integrate the eight predominant models at the time about technology acceptance. The UTAUT comprises eight constructs that influence the regular use of a technology, directly or indirectly. The selection of these frameworks as a starting point was guided by the authors’ previous experiences with the evaluation of medical informatics interventions.

### Literature Review

**Figure 2:**
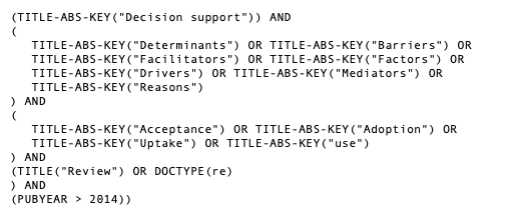
Search strategy used in Scopus.

Subsequent iterations comprised the mapping of constructs identified through a literature review of recent aggregative studies presenting determinants of the acceptance of CDSSs. To obtain the initial pool of references, we queried Scopus, MEDLINE (Medical Literature Analysis and Retrieval System Online), Embase, CINAHL (Cumulative Index of Nursing and Allied Health Literature), and PsycINFO. Figure 2 presents the search strategy used in Scopus. We constructed equivalent searches for the other databases.

Two investigators (JC and MZM) screened the initial reference pool and evaluated their titles and abstracts. For a reference to be selected, it needed to fulfill all of the following inclusion criteria: (1) address the topic of CDSSs, (2) employ literature review methods to obtain its results, and (3) include, among its results, determinants of the acceptance of CDSSs. Exclusion criteria were limited to the following: (1) the publication was not a research article (eg, abstract, viewpoint, commentary, editorial, or protocol) and (2) the full text was not in English or Spanish.

We limited the search strategy to articles published since 2014; we were working under the assumption that, although recent, the aggregate studies found would cover the relevant literature published before that year. That assumption was validated by documenting the period covered by each included review.

The investigators (JC and MZM) then extracted the constructs and their definitions from the full text of each article (see Multimedia Appendix 1). In cases where definitions or descriptions were not explicitly stated, the investigators reviewed the full text of the cited articles, including supplemental materials. Furthermore, in cases where other frameworks were used to organize the review findings, those frameworks were included in the mapping exercise with their own iterations.

### Domain Structure and Refinement

The grouping of constructs into domains was initially informed by the organization and definitions in the base frameworks (ie, TDF and UTAUT). Later on, new constructs identified from the literature reviews were contrasted with the domain definitions to choose their locations. In some cases, this process resulted in the creation of new domains or in changing previous definitions.

Finally, preliminary versions of the framework were discussed with the other authors (ZLL, SLKG, and RDB) and other colleagues, resulting in the refinement of construct labels, definitions, and grouping.

## Results

### Overview

**Figure 3:**
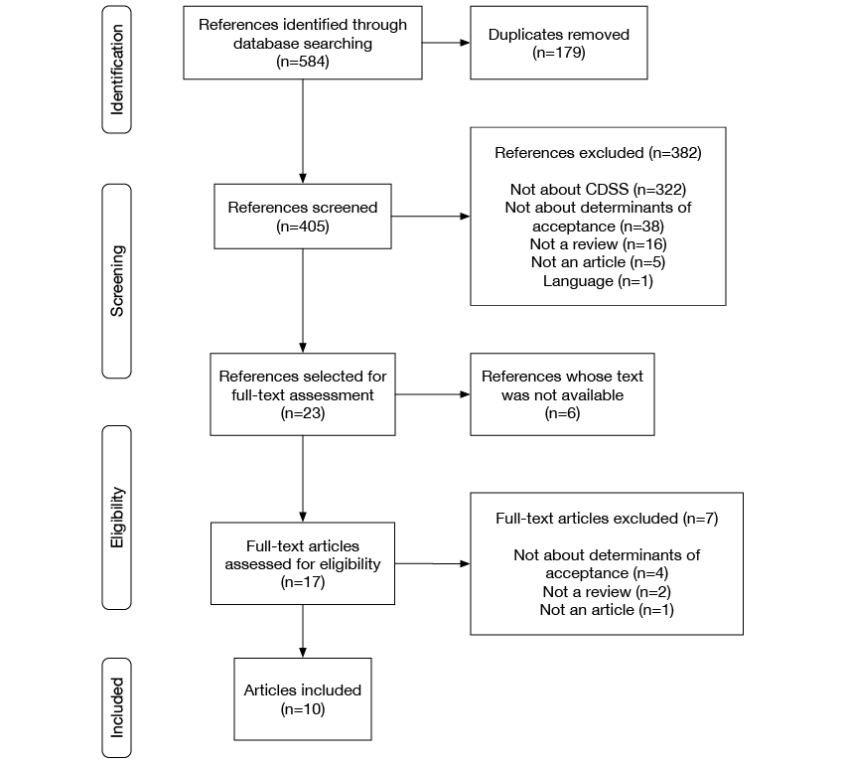
Flow diagram of the literature review. CDSS: clinical decision support system.

The mapping process comprised 13 iterations, corresponding to 10 reviews and four frameworks. The initial literature search identified 584 references. After removing duplicates, 405 references passed through the screening process (see Figure 3). Of these, 23 were selected for full-text assessment; however, text from six of these articles was not available. After reviewing the 17 available articles, seven were excluded. Finally, 10 articles met all inclusion criteria [35-40,42,45]. Only one article was excluded based on language. Table 1 shows the characteristics of the reviews included in this study and any associated frameworks [29,35-45].

### Included Reviews

The 10 reviews included in the mapping exercise (see Table 1) synthesize 219 studies from 1995 to 2018; these studies include participants from several populations of health professionals (ie, nurses, general practitioners, specialists, pharmacists, residents, laboratory technicians, physical therapists, medical assistants, medical students, paramedics, psychologists, and social workers). Additionally, two frameworks were included: the CFIR (Consolidated Framework for Implementation Research) [29], which was used in Ross et al [37], and the HOT-fit (Human, Organization, and Technology-ﬁt) framework [41], which was used in Kilsdonk et al [38] and Van Dort et al [44].

**Table 1:** Characteristics of included reviews.

Source	Number of references	Time span	Participants	Framework
Khong et al, 2015 [35]	16	2005-2014	Nurses, general practitioners, specialists, pharmacists, and medical assistants	N/A^a^
Khairat et al, 2018 [36]	14	1995-2015	Nurses, general practitioners, specialists, residents, and medical students	N/A
Ross et al, 2016 [37]	44	2002-2014	Nurses, general practitioners, specialists, laboratory technicians, physical therapists, paramedics, medical students, residents, pharmacists, and social workers	CFIR^b^ [29]
Kilsdonk et al, 2017 [38]	35	2003-2015	Nurses, general practitioners, specialists, physical therapists, medical students, residents, pharmacists, and psychologists	HOT-fit^c^ [41]
Miller et al, 2017 [39]	14	2003-2015	Nurses, general practitioners, specialists, pharmacists, medical assistants, and residents	N/A
Borum, 2018 [40]	9	2011-2016	Nurses, general practitioners, specialists, and pharmacists	N/A
Baig et al, 2019 [42]	22	2014-2016	Nurses, general practitioners, and specialists	N/A
Carter et al, 2019 [43]	13	2014-2017	Nurses, midwives, nurse students, specialists, and community health workers	N/A
Van Dort et al, 2019 [44]	13	2009-2018	General practitioners and specialists	HOT-fit [41]
Hussain et al, 2019 [45]	39	2008-2017	Nurses, general practitioners, and specialists	N/A

^a^N/A: not applicable.

^b^CFIR: Consolidated Framework for Implementation Research.

^c^HOT-fit: Human, Organization, and Technology-fit.

### BEAR

Table 2 presents the constructs and domains included in the proposed framework [28,34,46-50]. The mapping of each construct into the sources is included in Multimedia Appendix 2. BEAR comprises 156 constructs arranged into 22 domains. Domain definitions are included in Table 2, whereas definitions for each construct are included in Multimedia Appendix 1.

**Table 2:** BEAR (BEhavior and Acceptance fRamework) constructs and domains.

Domain^a^	Domain definition	Constructs^b^
Knowledge	Awareness, understanding, or information about a subject that has been obtained by experience or study: based on [46]	Knowledge Knowledge of task environment Procedural knowledge Knowledge of the decision algorithm Knowledge of the patient’s condition Previous experience with decision support technology
Skills, ability, and competence	An ability or proficiency acquired through training and practice [28]: based on [47]	Skills, ability, and competence Computer and mobile device skill Interpersonal skills Skills development
Role and identity	A coherent set of behaviors and displayed personal qualities of an individual in a social or work setting [28]: based on [47]	Individual identity Professional identity Organizational commitment Professional boundaries Professional role Professional autonomy
Beliefs about capabilities	Acceptance of the truth, reality, or validity about an ability, talent, or facility that a person can put to constructive use [28]: based on [47]	Beliefs about capabilities Empowerment Perceived behavioral control Professional confidence Self-confidence Self-efficacy Self-esteem
Beliefs about consequences	Acceptance of the truth, reality, or validity about outcomes of a behavior in a given situation [28]: based on [47]	Beliefs about consequences Anticipated regret Outcome expectancies Beliefs that technology would disrupt the delivery of care Characteristics of outcome expectancies Concerns about liability and responsibility Concerns over patient privacy
Attitudes	Relatively enduring and general evaluations of an object, person, group, issue, or concept on a dimension ranging from negative to positive. Attitudes provide summary evaluations of target objects and are often assumed to be derived from specific beliefs, emotions, and past behaviors associated with those objects [48].	Attitudes Interest in technology Perceived uselessness Optimism Pessimism Unrealistic optimism Attitude toward practice guidelines
Contingencies	A conditional probabilistic relationship between two events. Contingencies may be arranged via dependencies or they may emerge by accident [28]: citing [47].	Contingencies Consequences Reinforcement Incentives Punishment Rewards Sanctions
Intentions	A conscious decision to perform a behavior; a resolve to act in a certain way or an impulse for purposeful action. In experiments, intention is often equated with goals defined by the task instruction [28]: citing [47].	Intentions Stability of intentions Stages of change—precontemplation Stages of change—contemplation Stages of change—preparation Stages of change—action Stages of change—maintenance
Goals	Mental representations of outcomes or end states that an individual wants to achieve [28]: based on [47]	Goals Goals—level of control (autonomous vs controlled) Goals—temporality (distal vs proximal) Target setting Goal priority Action planning Change plan
Memory, attention, and decision processes	The ability to retain information, focus selectively on aspects of the environment, and choose between two or more alternatives [28]: based on [47]	Memory Attention Attention control Decision process Cognitive overload and tiredness
Environmental context and resources	Any circumstance of a person’s situation or environment that discourages or encourages the development of skills and abilities, independence, social competence, and adaptive behavior [28]: based on [47]	Environmental context Resources Environmental stressors Organizational structure Organizational culture and climate Assessment—skills Assessment—knowledge Assessment—performance Person × environment interaction Salient events and critical incidents Time availability—patient care Time availability—learning Technical support Technical infrastructure Facilities Implementation climate Tension for change Access to information and knowledge about the intervention
Social influences	Those interpersonal processes that can cause individuals to change their thoughts, feelings, or behaviors [28]: based on [47] The degree to which an individual perceives that others important to him or her believe he or she should use the new system [34]	Social influences Alienation Group conformity Group identity Group norms Leadership Intergroup conflict Modelling Power Social comparisons Social norms Social pressure Social support
Emotions	A complex reaction pattern, involving experiential, behavioral, and physiological elements, by which the individual attempts to deal with a personally significant matter or event [28]: based on [47]	Emotions Affect Positive affect Negative affect Anxiety Burnout Depression Apprehension Fear Stress Frustration Uncertainty Dissatisfaction
Behavioral regulation	Anything aimed at managing or changing objectively observed or measured actions [28]: based on [47]	Behavioral regulation Breaking habit Self-monitoring
Intervention characteristics	Intervention attributes that facilitate or hinder its implementation. The intervention includes not only the system but also all processes and resources needed to deploy it.	Intervention characteristics Intervention source Adaptability Trialability Interoperability Implementation complexity Costs—initial Costs—recurrent Voluntariness of use
Performance expectancy	The degree to which an individual believes that using the system will help him or her to attain gains in job performance [34]	Performance expectancy Benefits for the patient Improved communication with other health professionals Improved access to knowledge Consistency of care Error prevention Time-saving Habituation
Effort expectancy	The effort an individual believes is required to implement or use the system	Effort expectancy Quality of the user interface Compatibility with the clinical workflow Access at the point of care Familiarization
Demographic characteristics	The characteristics of people who form a particular group, with reference to distribution, composition, or structure: based on [46,49]	Demographic characteristics Age Gender Professional experience Training level and educational level Nationality
System quality	The degree to which the information and functions provided by the system meet the user’s needs or expectations and give user satisfaction; the degree to which the system is free from deficiencies or defects: based on [50]	System quality System performance Output quality Output quality—accuracy Output quality—completeness Output quality—specificity Output quality—timeliness System reliability
Agreement with the decision algorithm	The degree to which the user agrees that the decision algorithm is a correct way to make the intended decision	Agreement with the decision algorithm Applicability to complex cases Evidence strength and quality
Patient–health professional relationship	The way the system affects the relationship between the health professional and the patient	Patient–health professional relationship Obtrusiveness Diminished eye contact Disruption of flow in conversation with the patient Knowledgeable image
Patient’s preferences	The way the patient’s preferences affect the health professional’s decision about using the system	Patient’s preferences Patient’s decision not to follow the recommendation

^a^The way we include references in this column seeks to help the reader trace back the origin of each definition. In cases where we use the same text from the source (ie, a textual citation), we only include the reference number. In cases where the source text was adapted, we precede the reference number with the phrase “based on.” In cases where the source is citing another source, we include a reference for the latter, preceded by the word “citing.” Finally, definitions without a reference were developed by the authors.

^b^Construct definitions are included in Multimedia Appendix 1.

## Discussion

### Principal Findings

Our objective was to develop a framework, grounded in the literature about determinants of behavioral change and technology acceptance, that would be useful to researchers investigating the implementation of CDSSs as a strategy to foster the uptake of evidence-based recommendations.

### Developing Strategy and Structure

The idea of BEAR originated in our search for a conceptual framework to guide our research in the use of CDSSs as a strategy to implement clinical practice guidelines. From the beginning, we realized that the effectiveness of such an approach would be mediated by aspects of behavioral change and technology acceptance. We found part of the guidance we were looking for in the TDF [28,30] and the UTAUT [34]. These frameworks provide constructs that address both aspects of the phenomenon, although at a higher level than the one we were seeking, particularly on the side of technology acceptance. For example, UTAUT includes the concept of *facilitating conditions*, defined as “the degree to which an individual believes that an organizational and technical infrastructure exists to support the use of the system” [34]. However, that definition is not enough to identify what specific facilitating conditions are missing, which we consider a necessary step for the development of interventions. This need for further detail is what led us to an iterative process by which we incorporated the findings of recent literature reviews about determinants of the acceptance of CDSSs.

To capture the variability in the phenomenon of implementing CDSSs, we sought to include constructs that are specific enough to facilitate the identification of what is different between one implementation experience and another. For example, we added constructs to represent four quality aspects for the system’s output: accuracy, completeness, specificity, and timeliness. However, recognizing that it is unlikely that we have identified every relevant concept, we also included general constructs, in this case *output quality*. Along the same lines, most domains include a construct that shares the domain’s label. In cases where a domain label corresponds to a group of concepts (eg, *memory, attention, and decision processes*), we do not include a general construct but only those representing each constituting concept.

The decision to include both specific and general constructs led us to make two more decisions about the framework’s structure: (1) to have only one grouping level (ie, domain and construct) and (2) to include each construct only once, inside the domain where, in our opinion, the construct fits better. With these decisions, we sought to control complexity while maintaining detail. The resulting domains sort the constructs thematically; that is, constructs included in a domain represent determinants that could influence the concept represented by the domain, instead of particularizations of that concept.

### Use Cases

BEAR is not a parsimonious framework. We believe this is both a strength and a limitation. On the one hand, we expect that the level of detail facilitates the identification of actionable determinants; on the other hand, using the whole framework could be difficult, particularly in quantitative-oriented projects. However, for most projects, using every construct in the framework is not necessary or even advisable.

Our recommendation is to use the framework at the domain level during the initial stages of research design, particularly when discussing scope. Later, in qualitative-oriented studies, BEAR could be utilized to develop data collection guides for interviews, focus groups, or observations. This could be done at the domain level, in the case of exploratory studies, or based on selected constructs. During analysis, BEAR could serve as an initial coding schema, either at the domain or construct levels, raising the researcher’s awareness of determinants and supporting the identification of categories.

In quantitative-oriented studies, besides informing decisions about scope and research questions, BEAR could support the search for theories and measurement tools. In both cases, we recommend reviewing the definitions and references provided in Multimedia Appendix 1.

In the next section, we present two examples of how we have used BEAR in our research.

### Use Example: A CDSS to Support Chronic Obstructive Pulmonary Disease Active Case-Finding

We are currently using BEAR in a qualitative-oriented project whose objective is to identify barriers and facilitators to the use of a CDSS to support the implementation of a case-finding recommendation included in the Colombian chronic obstructive pulmonary disease (COPD) clinical practice guideline.

According to the recommendation, suspicion of airflow obstruction could be established by the identification of specific risk factors and symptoms. Once the suspicion is established, spirometry should be ordered to confirm the limitation in the airflow [51].

In the study, we explained the recommendation to primary-level physicians and asked them to use a CDSS, implemented as a mobile app, during their patient encounters. The CDSS asked a series of questions about the patient’s clinical history and current symptoms. Using this information, the system applied a decision algorithm to establish the suspicion of COPD, in which case it recommended that the participant order a spirometry test to discard or confirm the diagnosis.

After 2 months using the system, we interviewed the participants to explore their experiences applying the recommendation and using the system. The guide used in these interviews was developed using BEAR at the domain level. Table 3 presents selected questions included in the guide. Designing the interview around BEAR’s domains allowed us to explore both the behavioral change and the technology acceptance aspects of the intervention.

**Table 3:** Example 1 questions.

Domain	Questions
Knowledge	Before this project, did you know about this recommendation? Given the information you had before, and what we have given you in the project, do you consider that you have all the information you need to carry out the screening?
Role and identity	Do you consider that screening for COPD^a^ cases is part of the primary care physician’s responsibility or should it be assigned to someone else?
Performance expectancy	Was the app useful in the process of implementing the recommendation? How did you use it?
Agreement with the decision algorithm	Can you think of anything that the ministry could change in the content of the recommendation to make it easier to meet the goal of detecting COPD cases early?

^a^COPD: chronic obstructive pulmonary disease.

Data collected in the interviews were analyzed thematically using BEAR’s constructs as the initial coding schema. During the initial analysis, the transcript below—adapted from the original data in Spanish—was coded under the following constructs: *patient–health professional relationship, diminished eye-contact,* and *patient’s preferences*.

...nowadays, we hardly see the patient, we are always [gestures representing the use of a keyboard]. We are all the time writing in the health record...In fact, some patients get upset. They complain that I do not look them in the eye. I try to look at them while writing in the computer, but I don’t have the ability yet.

Participant

Does this mean that they got upset when you used the application on your phone?

Interviewer

No, because I tell them, “Look, I am going to use this app to help in the diagnosis,” and I show them my phone.

Participant

Later in the analysis, the review of the content in these codes led to development of two categories: *perceived loss of attention* and *negotiating the use of the device with the patient*. The first category refers to the way the patient seems to interpret and resent that he has lost the physician’s attention when the latter is using the computer. The second category refers to the way physicians prevent complaints about themselves when using the phone during the encounter by telling the patients what they are using the phone for and sometimes including them in the process of using the system. The relationship between these categories allows us to recognize differential effects over the *patient–health professional relationship* of CDSSs implemented as mobile apps and as desktop applications.

### Use Example: Clinician Responsiveness to the CDSS in Clinical Practice

Alert fatigue is a common problem for clinicians who use technology designed to improve patient safety. Evidence-based strategies to overcome alert fatigue are lacking, especially in the intensive care unit (ICU). There is an evidence gap, as discussed in a recent review and guidance document [52].

The goal of the project in this use example is to provide effective strategies for the management of alert fatigue in the ICU. The behavioral change of interest is increasing clinician responsiveness to CDSS alerts provided during patient care (eg, ordering medications). Formative research needs to be completed to understand the barriers and facilitators to clinicians’ responsiveness to alerts. To meet these goals, a mixed methods approach was applied using a survey and in-depth interviews conducted with critical care clinicians. Questions were developed based on BEAR at the domain level. A sample of selected questions is provided in Table 4.

**Table 4:** Example 2 questions.

Domain	Questions
Skills, ability, and competence	Do you feel competent to respond to the alerts you are receiving?
Beliefs about consequences	What do you think will happen if you do not respond to alerts?
Social influences	How responsive are your peers to alerts?
Emotions	How frequently does receiving an alert lead to an evoked emotional response?
Behavioral regulation	What would encourage you to be more responsive to alerts?
Performance expectancy	To what extent are the alerts useful?
Effort expectancy	How easy is it to respond to the alerts?

### BEAR in Relation to Other Frameworks

Out of 122 constructs identified in the literature review, 52 (42.6%) mapped to the TDF (see Multimedia Appendix 2). This supports our initial assumption that, in the context of CDSSs, behavioral change and technology acceptance are interrelated. Since the TDF was selected as a source from the beginning, it is not a surprise that both its constructs and structure had a substantial influence on the resulting framework. However, the TDF’s constructs emerged from the mapping process with some modifications. In some cases, these changes corresponded to the integration of constructs (eg, the TDF’s *skills*, *ability*, and *competence*, whose definitions in the American Psychological Association Dictionary of Psychology [48] are similar). In other cases, we changed the construct definition to facilitate its interpretation in the context of clinical practice change. For instance, the TDF’s definition for modelling—“In developmental psychology the process in which one or more individuals or other entities serve as examples (models) that a child will copy” [28]—was changed to “The process in which one or more individuals or other entities serve as examples (models) for a person to copy.” We believe these alterations do not substantially change the meaning of the affected TDF constructs, but rather improve their applicability.

The CFIR [29] and HOT-fit framework [41] were also part of the mapping process. However, several of their constructs were not included in the resulting framework due to differences in the scope. Whereas BEAR deals with behavioral change and technology acceptance from the individual's perspective, the CFIR considers the implementation as a whole, integrating other perspectives (ie, those related to the organization, the government and health system, and the implementation project [29]). In some cases, those perspectives intersected. For example, the CFIR’s *inner setting—implementation climate* construct represents an organizational characteristic that influences the individual’s behavior. However, in other cases (eg, the CFIR’s *process—reflecting and evaluating* construct), we did not identify a direct influence over the individual. The same happened with the HOT-fit framework’s *organization* domain [41]. We recognize that the level of influence of a particular construct over the individual’s behavior could be a matter of debate—indeed, we had several discussions about it during the mapping process—thus, we used our better judgment. For information about the mapping of specific constructs, we refer the reader to Multimedia Appendix 2.

This is certainly not the first attempt to apply technology acceptance models in health care [31,53]. The majority of these attempts have tried to adapt the Technology Acceptance Model (TAM) [33], one of UTAUT’s eight contributing frameworks [34]. BEAR has similarities and differences with these works. On the one hand, BEAR attempts to cover a wide range of possible determinants, but it does not make statements about the magnitude of their influence on each other or the individual’s behavior. In other words, BEAR does not attempt to state a theory about technology acceptance. Instead, BEAR is meant as an exploratory tool that allows for the identification of determinants that could be articulated into hypotheses and potentially form the basis of interventions. We hope that the study of those hypotheses in different health care contexts results in a future theory that is able to explain and predict practice change in the context of CDSSs.

On the other hand, literature reviews have found that the TAM has low explanatory power in health care environments [31,53]. The authors of these studies attribute this lack of fit to professional differences between health professionals and other workers [53] and to the fact that the TAM does not completely incorporate the emotional and cultural aspects of health care decision making [31]. Our interpretation of these findings is that behavioral change determinants operate differently in health care in comparison to other work environments. If that is correct, it could be expected that bridging the gap between behavioral change and technology acceptance brings forward the missing pieces in the puzzle.

Finally, our objective of this study was to develop a conceptual framework, not a theory. A theory serves as an explanation of a phenomenon that in many cases allows for the prediction of outcomes. However, our objective was not to predict an outcome or the relative weight of each determinant in the explanation of an outcome, but to synthesize and organize all potential determinants reported in the reviewed literature. That is why we do not state any conclusion about the relationship between specific constructs, besides grouping them into domains to facilitate organization and presentation, nor their relative contribution to the success in the implementation of CDSSs. In this sense, BEAR is akin to other determinant frameworks [54], such as the TDF [28,30] or the CFIR [29], rather than a theory, such as the UTAUT [34].

## Appendix

Multimedia Appendix 1 Definitions of constructs in BEAR (BEhavior and Acceptance fRamework).

Multimedia Appendix 2 Mapping of constructs in BEAR (BEhavior and Acceptance fRamework).

## Abbreviations

BEAR: BEhavior and Acceptance fRamework
CDSS: clinical decision support system
CFIR: Consolidated Framework for Implementation Research
CINAHL: Cumulative Index of Nursing and Allied Health Literature
COPD: chronic obstructive pulmonary disease
HOT-fit: Human, Organization, and Technology-fit
ICU: intensive care unit
MEDLINE: Medical Literature Analysis and Retrieval System Online
TAM: Technology Acceptance Model
TDF: Theoretical Domains Framework
UTAUT: Unified Theory of Acceptance and Use of Technology
