# Supplementary material for: A Conceptual Framework to Study the Implementation of Clinical Decision Support Systems (BEAR): Literature Review and Concept Mapping
Source: J Med Internet Res. 2020 Aug 6;22(8):e18388. doi: 10.2196/18388 (PMC7441385; doi:10.2196/18388)
Supplement: Multimedia Appendix 1 [file jmir_v22i8e18388_app1.doc]

| Label | Definition |
| --- | --- |
| Knowledge [1–6] | Awareness, understanding, or information about a subject that has been obtained by experience or study. Based on [7] |
| Knowledge [1–6] | Awareness, understanding, or information about a subject that has been obtained by experience or study. Based on [7] |
| Knowledge of task environment [1] | Knowledge of the social and material context in which a task is undertaken. [1] based on [8] |
| Procedural knowledge [1] | Knowing how to do something. [1] citing [9] |
| Knowledge of the decision algorithm [3] | Knowledge about the decision algorithm and its application. Based on [10] |
| Knowledge of the patient’s condition [3,6] | Knowledge about the patient clinical history and health state. Based on [10] |
| Previous experience with decision support technology [3,5] | The knowledge or skill that an individual has obtained through previous use of decision support tools. Based on [7] |
| Skills, ability, and competence [1–6,11,12] | An ability or proficiency acquired through training and practice. [1] based on [8] |
| Skills, ability, and competence [1–6,11,12] | The proficiency that an individual demonstrates when performing a task.  Based on  Skill: an ability or proficiency acquired through training and practice. [13]  Ability: existing competence or skill to perform a specific physical or mental act. [13]  Competence: one’s developed repertoire of skills, especially as it is applied to a task or set of tasks. [13] |
| Interpersonal skills [1] | An aptitude enabling a person to carry on effective relationships with others, such as an ability to cooperate, to assume appropriate social responsibilities or to exhibit adequate flexibility. [1] citing [8] |
| Skills development[1] | The gradual acquisition or advancement through progressive stages of an ability or proficiency acquired through training and practice. [1] based on [8,14] |
| Computer and mobile device skill [3–6,11,12] | The proficiency that an individual demonstrates when using a computer or a mobile device. |
| Role and Identity [1–5,11,12,15,16] | A coherent set of behaviors and displayed personal qualities of an individual in a social or work setting. [1] based on [8] |
| Individual identity [1] | An individual's sense of self defined by a) a set of physical and psychological characteristics that is not wholly shared with any other person and b) a range of social and interpersonal affiliations (e.g., ethnicity) and social roles. [1] citing [8] |
| Professional identity [1,5,12] | The characteristics by which an individual is recognized relating to, connected with or befitting a particular profession. [1] based on [14] |
| Organizational commitment [1,2,4] | An employee's dedication to an organization and wish to remain part of it. Organizational commitment is often described as having both an emotional or moral element and a more prudent element. [1] citing [8] |
| Professional boundaries [1,4,5] | The bounds or limits relating to, or connected with a particular profession or calling. [1] based on [14] |
| Professional role [1,3,4,12] | The behavior considered appropriate for a particular kind of work or social position. [1] based on [14] |
| Professional autonomy [3–5,11,15,16] | The quality or state of being independent and self-directing, especially in making decisions, enabling professionals to exercise judgment as they see fit during the performance of their jobs. [17] |
| Beliefs about capabilities [1–4,6,12] | Acceptance of the truth, reality, or validity about an ability, talent, or facility that a person can put to constructive use. [1] based on [8] |
| Beliefs about capabilities [1–4,6,12] | Acceptance of the truth, reality, or validity about an ability, talent, or facility that a person can put to constructive use. [1] based on [8] |
| Empowerment [1,2,4] | The promotion of the skills, knowledge and confidence necessary to take great control of one's life as in certain educational or social schemes; the delegation of increased decision-making powers to individuals or groups in a society or organization. [1] citing [8] |
| Perceived behavioral control [1] | An individual's perception of the ease or difficulty of performing the behavior of interest. [1] citing [18] |
| Professional confidence [1,2,4,12] | An individual's belief in his or her repertoire of skills, and ability especially as it is applied to a task or set of tasks. [1] based on [14] |
| Self-confidence [1,3,6] | Self-assurance or trust in one's own abilities, capabilities and judgment. [1] citing [8] |
| Self-efficacy [1,2,4] | An individual's capacity to act effectively to bring about desired results, as perceived by the individual. [1] based on [8] |
| Self-esteem [1] | The degree to which the qualities and characteristics contained in one's self-concept are perceived to be positive. [1] citing [8] |
| Beliefs about consequences [1,2,4,5,11,12,16,19] | Acceptance of the truth, reality, or validity about outcomes of a behavior in a given situation. [1] based on [8] |
| Beliefs about consequences [1,2,4,5,11,12,16,19] | Acceptance of the truth, reality, or validity about outcomes of a behavior in a given situation. [1] based on [8] |
| Anticipated regret [1] | A sense of the potential negative consequences of a decision that influences the choice made: for example, an individual may decide not to make an investment because of the feelings associated with an imagined loss. [1] citing [8] |
| Outcome expectancies [1] | Cognitive, emotional, behavioral, and affective outcomes that are assumed to be associated with future or intended behaviors. These assumed outcomes can either promote or inhibit future behaviors. [1] based on [8] |
| Beliefs that technology would disrupt the delivery of care [4,5,12,16] | The mental association of the introduction of new technology with potential adverse effects over the care processes. Based on [13] |
| Characteristics of outcome expectancies [1] | Characteristics of the cognitive, emotional and behavioral outcomes that individuals believe are associated with future or intended behaviors and that are believed to either promote or inhibit these behaviors. These include whether they are sanctions/rewards, proximal/distal, valued/not valued, probable/improbable, salient/not salient, perceived risks or threats. [1] based on [8] |
| Concerns about liability and responsibility [4,16] | Concerns related to the fact that someone is legally or organizationally responsible for something. Based on [7] |
| Concerns over patient privacy [4,11,19] | Concerns related to the fact that patient private information is made available or disclosed to unauthorized individuals. Based on [20] |
| Attitudes [1,3–5,12,16] | Relatively enduring and general evaluations of an object, person, group, issue, or concept on a dimension ranging from negative to positive. Attitudes provide summary evaluations of target objects and are often assumed to be derived from specific beliefs, emotions, and past behaviors associated with those objects. [13] |
| Attitudes [1,3–5,12,16] | Relatively enduring and general evaluations of an object, person, group, issue, or concept on a dimension ranging from negative to positive. Attitudes provide summary evaluations of target objects and are often assumed to be derived from specific beliefs, emotions, and past behaviors associated with those objects. [13] |
| Interest in technology [4] | An attitude characterized by a need or desire to give selective attention to new machinery and equipment. Based on [7,13] |
| Perceived uselessness [4,12,16] | An attitude characterized by doubts that the intervention will improve patient care. Based on [4] |
| Optimism [1,3,12] | The attitude that outcomes will be positive and that people's wishes or aims will ultimately be fulfilled. [1] based on [8] |
| Pessimism [1,5] | The attitude that things will go wrong and that people's wishes or aims are unlikely to be fulfilled. [1] citing [8] |
| Unrealistic optimism [1] | The inert tendency for humans to over-rate their own abilities and chances of positive outcomes compared to those of other people. [1] based on [21] |
| Attitude toward practice guidelines [5] | A general evaluation of the usefulness or convenience of having practice guidelines. Based on [13] |
| Contingencies [1,2,4] | A conditional probabilistic relation between two events. Contingencies may be arranged via dependencies or they may emerge by accident. [1] citing [8] |
| Contingencies [1,2,4] | A conditional probabilistic relation between two events. Contingencies may be arranged via dependencies or they may emerge by accident. [1] citing [8] |
| Reinforcement [1] | A process in which the frequency of a response is increased by a dependent relationship or contingency with a stimulus. [1] based on [8] |
| Consequences [1] | An outcome of behavior in a given situation. [1] citing [8] |
| Incentives [1,2,4] | An external stimulus, such as condition or object, that enhances or serves as a motive for behavior. [1] citing [8] |
| Punishment [1] | The process in which the relationship between a response and some stimulus or circumstance results in the response becoming less probable; a painful, unwanted or undesired event or circumstance imposed as a penalty on a wrongdoer. [1] citing [8] |
| Rewards [1,2,4] | Return or recompense made to, or received by a person contingent on some performance. [1] based on [14] |
| Sanctions [1] | A punishment or other coercive measure, usually administered by a recognized authority, that is used to penalize and deter inappropriate or unauthorized actions. [1] citing [8] |
| Intentions [1–5] | A conscious decision to perform a behavior; a resolve to act in a certain way or an impulse for purposeful action. In experiments, intention is often equated with goals defined by the task instruction. [1] citing [8] |
| Intentions [1–5] | A conscious decision to perform a behavior; a resolve to act in a certain way or an impulse for purposeful action. In experiments, intention is often equated with goals defined by the task instruction. [1] citing [8] |
| Stability of intentions [1] | Ability of one's resolve to remain in spite of disturbing influences. [1] based on [14] |
| Stages of change – Precontemplation [1–5] | ﻿Stages in the Transtheoretical Model of Health Behavior  Change, which proposes that behavior change is accomplished through five specific stages: Precontemplation, Contemplation, Preparation, Action, and Maintenance. Based in [1], based on [22]  Precontemplation is the stage in which people are not intending to take action in the foreseeable future, usually measured as the next 6 months. [23] |
| Stages of change – Contemplation [1–5] | ﻿Stages in the Transtheoretical Model of Health Behavior  Change, which proposes that behavior change is accomplished through five specific stages: Precontemplation, Contemplation, Preparation, Action, and Maintenance. Based in [1], based on [22]  ﻿Contemplation is the stage in which people are intending to change in the next 6 months. They are more aware of the pros of changing but are also acutely aware of the cons. [23] |
| Stages of change – Preparation [1–5] | ﻿Stages in the Transtheoretical Model of Health Behavior  Change, which proposes that behavior change is accomplished through five specific stages: Precontemplation, Contemplation, Preparation, Action, and Maintenance. Based in [1], based on [22]  ﻿Preparation is the stage in which ﻿people are intending to take action in the immediate future, usually measured as the next month. They have typically taken some significant action in the past year. These individuals have a plan of action, ﻿such as joining a health education class, consulting a counselor, talking to their physician, buying a self-help book, or relying on a self-change approach. [23] |
| Stages of change – Action [1–5] | ﻿Stages in the Transtheoretical Model of Health Behavior  Change, which proposes that behavior change is accomplished through five specific stages: Precontemplation, Contemplation, Preparation, Action, and Maintenance. Based in [1], based on [22]  ﻿Action is the stage in which people have made specific overt modifications in their life styles within the past 6 months. [23] |
| Stages of change – Maintenance [1–5] | ﻿Stages in the Transtheoretical Model of Health Behavior  Change, which proposes that behavior change is accomplished through five specific stages: Precontemplation, Contemplation, Preparation, Action, and Maintenance. Based in [1], based on [22]  ﻿﻿Maintenance is the stage in which people are working to prevent relapse, but they do not apply change processes as frequently as do people in action. They are less tempted to relapse and increasingly more confident that they can continue their changes. [23] |
| Goals [1–6] | Mental representations of outcomes or end states that an individual wants to achieve. [1] based on [8] |
| Goals [1–5] | Mental representations of outcomes or end states that an individual wants to achieve. [1] based on [8] |
| Goals - Level of control (autonomous vs. controlled) [1,2,4,5] | The degree to which an individual’s goals are set by himself or by an external entity. Based on [13] |
| Goals - Temporality (distal vs. proximal) [1] | The degree to which an individual’s goals are closer (proximal) or further away (distal). Based on [1] |
| Target setting [1,2,4] | A process that establishes specific time-based behavior targets that are measurable, achievable and realistic. [1] citing [8] |
| Goal priority [1–4] | Order of importance or urgency of end states toward which one is striving. [1] based on [8] |
| Action planning [1,6] | The action or process of forming a plan regarding a thing to be done or a deed. [1] based on [14] |
| Change plan [1] | The plan that one creates in advance of when, where and how one will enact a behavior. [1] based on [24] |
| Memory, Attention, and Decision Processes [1,5] | The ability to retain information, focus selectively on aspects of the environment and choose between two or more alternatives. [1] based on [8] |
| Memory [1,5] | The ability to retain information or a representation of a past experience, based on the mental processes of learning or encoding retention across some interval of time, and retrieval or reactivation of the memory; specific information of a specific past. [1] citing [8] |
| Attention [1,5] | A state of awareness in which the senses are focused selectively on aspects of the environment and the central nervous system is in a state of readiness to respond to stimuli. [1] citing [8] |
| Attention control [1,5] | The extent to which a person can concentrate on relevant cues and ignore all irrelevant cues in a given situation. [1] based on [8] |
| Decision process [1] | The cognitive process of choosing between two or more alternatives, ranging from the relatively clear cut to the complex. [1] citing [8] |
| Cognitive overload / tiredness [1,5] | The situation in which the demands placed on a person by mental work are greater than a person's mental abilities. [1] citing [8] |
| Environmental Context and Resources [1–6,11,12,15,16,19,25,26] | Any circumstance of a person's situation or environment that discourages or encourages the development of skills and abilities, independence, social competence, and adaptive behavior. [1] based on [8] |
| Environmental Context [1,5] | Any circumstance of a person's situation or environment that discourages or encourages the development of skills and abilities, independence, social competence, and adaptive behavior. [1] based on [8] |
| Resources [1,2,4,5] | A useful or valuable possession or quality that a person or organization has, for example, money, time, or skills. Based on [7] |
| Environmental stressors [1,5] | External factors in the environment that cause stress. [1] based on [8] |
| Organizational structure [2–4,11,16] | A tool and instrument used by enterprises to achieve their objectives and to dominate and coordinate decision-making activities. It may include type and size (e.g., number of beds), culture, politics, hierarchy, autonomy, planning and control systems, strategy, management and communications, leadership, top management support, and medical staff sponsorship. [27] citing [11,28] |
| Organizational culture / climate [1,2,4,5,11,16] | A distinctive pattern of thought and behavior shared by members of the same organization and reflected in their language, values, attitudes, beliefs and customs. [1] citing [8] |
| Assessment – Skills [1] | A judgment of the quality, worth, importance, or value of an individual’s skills. Based on [13] |
| Assessment – Knowledge[[1]](#footnote-2) | A judgment of the quality, worth, importance, or value of an individual’s knowledge. Based on [13] |
| Assessment – Performance[[2]](#footnote-3) | A judgment of the quality, worth, importance, or value of an individual’s performance. Based on [13] |
| Person x environment interaction [1,2,4] | Interplay between the individual and their surroundings. [1] |
| Salient events / critical incidents [1] | Occurrences that one judges to be distinctive, prominent or otherwise significant. [1] based on [8] |
| Time availability – patient care [3,5,12] | The amount of time that the health professional has to perform the tasks included in the care processes. |
| Time availability – learning [4] | The amount of time that the health professional has to acquire the knowledge and skills required to change his/her practice. |
| Technical support [3–5,11,16,19] | The availability of technical advice and help to facilitate the use of the system. Based on [7] |
| Technical infrastructure [3–6,19,25,26] | The degree to which an organizational and technical infrastructure exists to support the use of the system. Based on [26] |
| Facilities [3] | The degree to which the availability of facilities allows or hinders using the system or following the recommendation. Based on [29] |
| Implementation climate [2,4,16] | The shared receptivity of involved individuals to an intervention and the extent to which use of that intervention will be “rewarded, supported, and expected within their organization” [2] citing [30,31] |
| Tension for change [2,4,16] | The degree to which stakeholders perceive the current situation as intolerable or needing change. [2] citing [31–34] |
| Access to information and knowledge about the intervention [2–6,11,15,16,25] | Ease of access to digestible information and knowledge about the intervention and how to incorporate it into work tasks. [2] citing [31,35–37] Strategies to facilitate this access could include: printed educational materials, online resources, training, etc. |
| Social influences [1–5,11,15,16,26] | Those interpersonal processes that can cause individuals to change their thoughts, feelings, or behaviors. [1] based on [8]  The degree to which an individual perceives that important others believe he or she should use the new system. [26] |
| Social influences [1–5,11,15,16,26] | Those interpersonal processes that can cause individuals to change their thoughts, feelings, or behaviors. [1] based on [8]  The degree to which an individual perceives that important others believe he or she should use the new system. [26] |
| Alienation [1] | Estrangement from one's social group; a deep-seated sense of dissatisfaction with one's personal experiences that can be a source of lack of trust in one's social or physical environment or in oneself; the experience of separation between thoughts and feelings. [1] citing [8] |
| Group conformity [1,4] | The act of consciously maintaining a certain degree of similarity to those in your general social circles. [1] based on [24] |
| Group identity [1,2,4] | The set of behavioral or personal characteristics by which an individual is recognizable [and portrays] as a member of a group. [1] based on [24] |
| Group norms [1] | Any behavior, belief, attitude or emotional reaction held to be correct or acceptable by a given group in society. [1] based on [14] |
| Leadership [1,3,11,16] | The processes involved in leading others, including organizing, directing, coordinating and motivating their efforts toward achievement of certain group or organization goals. [1] citing [8] |
| Intergroup conflict [1] | Disagreement or confrontation between two or more groups and their members. This may involve physical violence, interpersonal discord, or psychological tension. [1] citing [38] |
| Modelling [1,11] | The process in which one or more individuals or other entities serve as examples (models) for a person to copy. |
| Power [1] | The capacity to influence others, even when they try to resist this influence. [1] citing [8] |
| Social comparisons [1,4,5] | The process by which people evaluate their attitudes, abilities, or performance relative to others. [1] based on [39] |
| Social norms [1,15,16] | Socially determined consensual standards that indicate a) what behaviors are considered typical in a given context and b) what behaviors are considered proper in the context. [1] citing [8] |
| Social pressure [1,16] | The exertion of influence on a person or group by another person or group. [1] citing [8] |
| Social support [1] | The apperception or provision of assistance or comfort to others, typically in order to help them cope with a variety of biological, psychological and social stressors. Support may arise from any interpersonal relationship in an individual's social network, involving friends, neighbors, religious institutions, colleagues, caregivers or support groups. [1] based on [8] |
| Emotions [1–4,6] | A complex reaction pattern, involving experiential, behavioral, and physiological elements, by which the individual attempts to deal with a personally significant matter or event. [1] based on [8] |
| Emotions [1–4,6] | A complex reaction pattern, involving experiential, behavioral, and physiological elements, by which the individual attempts to deal with a personally significant matter or event. [1] based on [8] |
| Affect [1] | An experience or feeling of emotion, ranging from suffering to elation, from the simplest to the most complex sensations of feelings, and from the most normal to the most pathological emotional reactions. [1] citing [8] |
| Positive affect [1,2,4,6] | The internal feeling/state that occurs when a goal has been attained, a source of threat has been avoided, or the individual is satisfied with the present state of affairs. Based on [1] |
| Negative affect [1,2,4] | The internal feeling/state that occurs when a goal has not been attained, a source of threat has not been avoided, or the individual is not satisfied with the present state of affairs. Based on [1] |
| Anxiety [1] | A mood state characterized by apprehension and somatic symptoms of tension in which an individual anticipates impending danger, catastrophe or misfortune. [1] citing [8] |
| Burn-out [1] | Physical, emotional or mental exhaustion, especially in one's job or career, accompanied by decreased motivation, lowered performance and negative attitudes towards oneself and others. [1] citing [8] |
| Depression [1] | A mental state that presents with depressed mood, loss of interest or pleasure, feelings of guilt or low self-worth, disturbed sleep or appetite, low energy, and poor concentration. [1] citing [40] |
| Apprehension [3] | Uneasiness or dread about an upcoming event or the future generally. [13] |
| Fear [1,4] | An intense emotion aroused by the detection of imminent threat, involving an immediate alarm reaction that mobilizes the organism by triggering a set of physiological changes. [1] citing [8] |
| Stress [1] | A state of physiological or psychological response to internal or external stressors. [1] citing [8] |
| Frustration [3] | The thwarting of impulses or actions that prevents individuals from obtaining something they have been led to expect based on past experience, as when a hungry animal is prevented from obtaining food that it can see or smell or when a child is prevented from playing with a visible toy. Internal forces can include motivational conflicts and inhibitions; external forces can include the actions of other individuals, admonitions of parents or others, and the rules of society. [13] |
| Uncertainty [3,4] | The state or condition in which something (e.g., the probability of a particular outcome) is not accurately or precisely known. [13] |
| Dissatisfaction [3,4] | Being not pleased with something; feeling that something is not as good as it should be. Based on [7] |
| Behavioral Regulation [1,15] | Anything aimed at managing or changing objectively observed or measured actions. [1] based on [8] |
| Behavioral Regulation [1,15] | Anything aimed at managing or changing objectively observed or measured actions. [1] based on [8] |
| Breaking habit [1] | To discontinue a behavior or sequence of behaviors that is automatically activated by relevant situational cues. [1] based on [8] |
| Self-monitoring [1,15] | A method used in behavioral management in which individuals keep a record of their behavior, especially in connection with efforts to change or regulate the self; a personality trait reflecting an ability to modify one's behavior in response to situation. [1] citing [8] |
| Intervention characteristics [2–6,11,12,15,16,19,25,26,41] | Intervention attributes that facilitate or hinder its implementation. The intervention includes not only the system but also all processes and resources needed to deploy it. |
| Intervention characteristics [2–6,11,12,15,16,19,25,26,41] | Intervention attributes that facilitate or hinder its implementation. The intervention includes not only the system but also all processes and resources needed to deploy it. |
| Intervention source [2,4–6,15,16,41] | Perception of key stakeholders about whether the intervention is externally or internally developed. [2] citing [31,42] |
| Adaptability [2,4–6,11,15,19] | The degree to which an intervention can be adapted, tailored, refined, or reinvented to meet local needs. [2] |
| Trialability [2,4,5] | The ability to test the intervention on a small scale in the organization, and to be able to reverse course (undo implementation) if warranted. [2] citing [31,43] |
| Interoperability [4–6,12,15,19,25] | The ability of two or more systems or elements to exchange information and to use the information that has been exchanged. [20] |
| Implementation complexity [2,4,19] | Perceived difficulty of implementation, reflected by intervention type (e.g., behavior change or plug-in technology), duration, scope, radicalness, disruptiveness, centrality, intricacy, and number of steps required to implement. [2] citing [31,44,45] |
| Costs - Initial [2,4,6,19] | The amount of money required to start using the system. Based on [7] |
| Costs - Recurrent [2,4,19] | The amount of money paid recurrently to use the system. Based on [7] |
| Voluntariness of use [3,5,26] | The degree to which the individual is free to choose using or not the technology without compulsion or obligation. Based on [46] |
| Performance expectancy [2–6,11,12,15,16,19,25,26,41] | The degree to which an individual believes that using the system will help him or her to attain gains in job performance. [26] |
| Performance expectancy [2–6,11,12,15,16,19,25,26,41] | The degree to which an individual believes that using the system will help him or her to attain gains in job performance. [26] |
| Benefits for the patient [4–6,11,12,16] | The mental association of the use of the system with potential benefits to the patient’s health or well-being. Based on [13] |
| Improved communication with other health professionals [3,6,11,15] | The degree to which the system improves the communication between members of the clinical team. Based on [47] |
| Improved access to knowledge [5,6,12,15,19] | The degree to which the system improves the access to knowledge. |
| Consistency of care [3,12,15] | The degree to which clinical practice is free from unnecessary variations across health professionals and patients. |
| Error prevention [5,11,15,16] | The degree to which an individual believes that using the system would help him to prevent making errors. |
| Timesaving [5,6,12,15,16] | The degree to which using the system reduces the time needed to perform a task. Based on [7] |
| Habituation [5,16,25,41] | The diminished effectiveness of a stimulus in eliciting a response, following repeated exposure to the stimulus. [13] A decrease in the perception of usefulness related to the user growing accustomed to the recommendations or alerts provided by the system. |
| Effort expectancy [2–6,11,12,15,16,19,25,26,41] | The effort an individual believes is required to implement or use the system. |
| Effort expectancy [2–6,11,12,15,16,19,25,26,41] | The effort an individual believes is required to implement or use the system. |
| Quality of the user interface [3,4,41,5,6,11,12,15,16,19,25] | The degree to which the user interface (the part of the application that permits the user and the application to communicate with each other) is free from deficiencies or defects. Based on [20] |
| Compatibility with the clinical workflow [2,3,25,41,4–6,11,12,15,16,19] | The degree to which the system fits with the care processes facilitating the work of the health personnel. Based on [3] |
| Access at the point of care [4,5,15,19] | The ability of the system to be used at the time and place of the patient care. |
| Familiarization [5,6] | To make acquainted or skilled, by practice or study. Based on [48]. A decrease in the perception of the effort needed to use the system related to the user growing accustomed or skilled. |
| Demographic characteristics [3–5,12,26] | The characteristics of people who form a particular group, with reference to distribution, composition, or structure. Based on [7,17] |
| Demographic characteristics [3–5,12,26] | The characteristics of people who form a particular group, with reference to distribution, composition, or structure. Based on [7,17] |
| Age [3–5,12,26] | The amount of time that has passed since an organism’s birth. [13] |
| Gender [4,26] | The condition of being male, female, or neuter. In a human context, the distinction between gender and sex reflects the usage of these terms: Sex usually refers to the biological aspects of maleness or femaleness, whereas gender implies the psychological, behavioral, social, and cultural aspects of being male or female (i.e., masculinity or femininity). [13] |
| Professional experience [3–5,26] | The knowledge or skill that an individual has obtained from doing a job. Based on [7] |
| Training level / Educational level [3–5] | The level of education or training attained by an individual. |
| Nationality [4] | The status of belonging to a particular nation by birth or naturalization. [49] |
| System quality [2,3,41,4,5,11,12,15,16,19,25] | The degree to which the information and functions provided by the system meet the user’s needs or expectations and give user satisfaction. The degree to which the system is free from deficiencies or defects. Based on [50] |
| System quality [2,3,41,4,5,11,12,15,16,19,25] | The degree to which the information and functions provided by the system meet the user’s needs or expectations and give user satisfaction. The degree to which the system is free from deficiencies or defects. Based on [50] |
| System performance [4,5,11,12,15,19] | A measure of a computer system or subsystem to perform its functions; for example, response time, throughput, or number of transactions per second. The efficiency of a system in accomplishing pieces of work is an attribute of performance. [20] |
| Output quality [3–5,11] | The degree to which the information presented by the system is free from deficiencies or defects. Based on [50] |
| Output quality – Accuracy [4,5,11,12,16,19] | The degree to which the information presented by the system corresponds to a correct application of the decision algorithm. |
| Output quality – Completeness [5,11,15,19] | The degree to which the system provides all the information required by the user to make the intended decision or to perform the intended behavior. |
| Output quality – Specificity [5,11,12,15,16,19,25,41] | The degree to which the system provides only the information required by the user to make the intended decision or to perform the intended behavior. The degree to which the system output is free from unnecessary information. |
| Output quality – Timeliness [5,11,12,15,19,25] | The degree to which the information presented by the system is available at the time is needed and based in up-to-date input. |
| System reliability [3–5,11,15,19] | The degree to which the system continually performs as the user expects, including –but not limited to– being available when the user tries to use it, and not presenting faults. Based on [7] |
| Agreement with the decision algorithm [2–5,11,12,15,16,25] | The degree to which the user agrees that the decision algorithm is a correct way to make the intended decision. |
| Agreement with the decision algorithm [2–5,11,12,15,16,25] | The degree to which the user agrees that the decision algorithm is a correct way to make the intended decision. |
| Applicability to complex cases [3,5,12,15,16] | The degree to which the user agrees that the decision algorithm applies to cases whose complexity or difficulty exceeds the average. |
| Evidence strength & quality [2,4,5,12,15,16,25] | Stakeholders’ perceptions of the quality and validity of evidence supporting the belief that the intervention will have desired outcomes. [2] |
| Patient-health professional relationship [3–6,12,19] | The way the system affects the relation between the health professional and the patient. |
| Patient-health professional relationship [3–6,12,19] | The way the system affects the relation between the health professional and the patient. Based on [3] |
| Obtrusiveness [3] | The degree to which the system blocks or gets in the way of the relation between the health professional and the patient. Based on [7] |
| Diminished eye-contact [3] | The degree to which the system gets in the way of the health professional and the patient looking at each other's eyes. Based on [7] |
| Disruption of flow in conversation with the patient [3,5] | The degree to which the system gets in the way of an effort-less flow in the conversation between the health professional and the patient. Based on [51] |
| Knowledgeable image [3,12] | The degree to which using the system affects the appraisal, the patient does, about the knowledge of the health professional. Based on [52] |
| Patient’s preferences [2–5,15] | The way the patient's preferences affect the health professional decision about using the system. |
| Patient’s preferences [2–5,15] | The way the patient's preferences affect the health professional decision about using the system. |
| Patient’s decision not to follow the recommendation [3] | The way the patient's decision not to follow the recommendation affects the health professional decision of using the system. For instance, a rejection from the patient to an intervention could make the system irrelevant in future encounters. |

**References**

1. Cane J, O’Connor D, Michie S. Validation of the theoretical domains framework for use in behaviour change and implementation research. Implement Sci. 2012 Jan;7:37.

2. Damschroder LJ, Aron DC, Keith RE, Kirsh SR, Alexander J a, Lowery JC. Fostering implementation of health services research findings into practice: a consolidated framework for advancing implementation science. Implement Sci. 2009;4(50):40–55.

3. Khong PCB, Holroyd E, Wang W. A critical review of the theoretical frameworks and the conceptual factors in the adoption of clinical decision support systems. CIN - Comput Informatics Nurs. 2015;33(12):555–70.

4. Ross J, Stevenson F, Lau R, Murray E. Factors that influence the implementation of e-health: a systematic review of systematic reviews (an update). Implement Sci. 2016 Dec 26;11(1):146.

5. Kilsdonk E, Peute LW, Jaspers MWM. Factors influencing implementation success of guideline-based clinical decision support systems: A systematic review and gaps analysis. Int J Med Inform. 2017 Feb 1;98:56–64.

6. Carter J, Sandall J, Shennan AH, Tribe RM. Mobile phone apps for clinical decision support in pregnancy: a scoping review. BMC Med Inform Decis Mak. 2019 Nov 12;19(1):219.

7. Cambridge Dictionary [Internet]. [cited 2019 Apr 27]. Available from: https://dictionary.cambridge.org/

8. American Psychological Association. APA Dictionary of Psychology. VandenBos GR, editor. Washington, DC: American Psychological Association APA; 2007.

9. Sutherland S. The Macmillan Dictionary of Psychology. 2nd ed. The Macmillan Dictionary of Psychology. London: Palgrave Macmillan; 1995.

10. Dowding D, Mitchell N, Randell R, Foster R, Lattimer V, Thompson C. Nurses’ use of computerised clinical decision support systems: a case site analysis. J Clin Nurs. 2009 Apr 1;18(8):1159–67.

11. Yusof MM, Kuljis J, Papazafeiropoulou A, Stergioulas LK. An evaluation framework for Health Information Systems: human, organization and technology-fit factors (HOT-fit). Int J Med Inform. 2008 Jun 1;77(6):386–98.

12. Khairat S, Marc D, Crosby W, Al Sanousi A. Reasons for physicians not adopting clinical decision support systems: Critical analysis. Journal of Medical Internet Research. 2018.

13. American Psychological Association. APA Dictionary of Psychology [Internet]. [cited 2019 Apr 24]. Available from: https://dictionary.apa.org/

14. Oxford English Dictionary Online [Internet]. Available from: http://www.oed.com/

15. Miller K, Mosby D, Capan M, Kowalski R, Ratwani R, Noaiseh Y, Kraft R, Schwartz S, Weintraub WS, Arnold R. Interface, information, interaction: a narrative review of design and functional requirements for clinical decision support. J Am Med Informatics Assoc. 2017;25(5):585–92.

16. Van Dort BA, Zheng WY, Baysari MT. Prescriber perceptions of medication-related computerized decision support systems in hospitals: A synthesis of qualitative research. Int J Med Inform. 2019;129(June):285–95.

17. MeSH - Medical Subject Headings. U.S. National Library of Medicine;

18. Ajzen I. The theory of planned behavior. Organ Behav Hum Decis Process. 1991 Dec;50(2).

19. Baig MM, GholamHosseini H, Moqeem AA, Mirza F, Lindén M. Clinical decision support systems in hospital care using ubiquitous devices: Current issues and challenges. Health Informatics J. 2019 Sep 17;25(3):1091–104.

20. IEEE. IEEE Standards Dictionary Online [Internet]. [cited 2019 May 14]. Available from: http://ieeexplore.ieee.org/xpls/dictionary.jsp

21. Ogden J. Health psychology : a textbook. 2nd ed. Open University Press; 2000. 396 p.

22. Prochaska JO, DiClemente CC. The Transtheoretical Approach: Crossing Traditional Boundaries of Change. Homewood, Ill. : Dow Jones-Irwin; 1984.

23. Prochaska J, Velicer W. The transtheoretical model of health behavior change. Am J Heal Promot. 1997;12(1):38–48.

24. Wikipedia, the free encyclopedia [Internet]. [cited 2019 Apr 25]. Available from: https://en.wikipedia.org/wiki/Main_Page

25. Borum C. Barriers for Hospital-Based Nurse Practitioners Utilizing Clinical Decision Support Systems: A Systematic Review. CIN Comput Informatics, Nurs. 2018;36(4):177–82.

26. Venkatesh V, Morris MG, Davis GB, Davis FD. User acceptance of information technology: toward a unified view. MIS Q. 2003 Sep 1;27(3):425–78.

27. Hsiao J-L, Wu W-C, Chen R-F. Factors of accepting pain management decision support systems by nurse anesthetists. BMC Med Inform Decis Mak. 2013 Dec 29;13(1):16.

28. Sciulli LM. How organizational structure influences success in various types of innovation. J Retail Bank Serv. 1998;20(1):13–9.

29. Goud R, van Engen-Verheul M, de Keizer NF, Bal R, Hasman A, Hellemans IM, Peek N. The effect of computerized decision support on barriers to guideline implementation: A qualitative study in outpatient cardiac rehabilitation. Int J Med Inform. 2010 Jun 1;79(6):430–7.

30. Klein KJ, Sorra JS. The challenge of innovation implementation. Acad Manag Rev. 1996;21(4):1055–80.

31. GREENHALGH T, ROBERT G, MACFARLANE F, BATE P, KYRIAKIDOU O. Diffusion of Innovations in Service Organizations: Systematic Review and Recommendations. Milbank Q. 2004 Dec 1;82(4):581–629.

32. Bodenheimer T, Wagner EH, Grumbach K. Improving Primary Care for Patients With Chronic Illness. JAMA. 2002 Oct 16;288(15):1909.

33. Bodenheimer T. Improving Primary Care for Patients With Chronic Illness. JAMA. 2002 Oct 9;288(14):1775.

34. Simpson DD, Dansereau DF. Assessing organizational functioning as a step toward innovation. Sci Pract Perspect. 2007 Apr;3(2):20–8.

35. Klein KJ, Conn AB, Sorra JS. Implementing computerized technology: An organizational analysis. J Appl Psychol. 2001;86(5):811–24.

36. Wallin L, Estabrooks CA, Midodzi WK, Cummings GG. Development and Validation of a Derived Measure of Research Utilization by Nurses. Nurs Res. 2006;55(3).

37. Helfrich CD, Weiner BJ, McKinney MM, Minasian L. Determinants of Implementation Effectiveness. Med Care Res Rev. 2007 Jun 5;64(3):279–303.

38. Forsyth DR. Group Dynamics. 5th ed. Vol. 84. Belmont: Wadsworth Cengage Learning; 2010.

39. Alicke MD. Evaluating Social Comparison Targets. In: Suls J, Wheeler L, editors. Handbook of Social Comparison: Theory and Research. New York: Plenum Publishing; 2000.

40. World Health Organization (WHO) [Internet]. Available from: www.who.int/mental_health/ management/depression/definition/en/

41. Hussain MI, Reynolds TL, Zheng K. Medication safety alert fatigue may be reduced via interaction design and clinical role tailoring: A systematic review. Journal of the American Medical Informatics Association. 2019.

42. Van de Ven AH. The innovation journey. Oxford ;;New York: Oxford University Press; 1999. 422 p.

43. Feldstein AC, Glasgow RE. A practical, robust implementation and sustainability model (PRISM) for integrating research findings into practice. Jt Comm J Qual patient Saf. 2008;

44. GROL RPTM, BOSCH MC, HULSCHER MEJL, ECCLES MP, WENSING M. Planning and Studying Improvement in Patient Care: The Use of Theoretical Perspectives. Milbank Q. 2007 Mar 1;85(1):93–138.

45. Gustafson DH, Sainfort F, Eichler M, Adams L, Bisognano M, Steudel H. Developing and Testing a Model to Predict Outcomes of Organizational Change. Health Serv Res. 2003 Apr 1;38(2):751–76.

46. Dictionary.com | Meanings and Definitions of Words at Dictionary.com [Internet]. [cited 2019 Apr 27]. Available from: https://www.dictionary.com/

47. Clarke HF, Bradley C, Whytock S, Handfield S, van der Wal R, Gundry S. Pressure ulcers: implementation of evidence-based nursing practice. J Adv Nurs. 2005 Mar 1;49(6):578–90.

48. Power Thesaurus [Internet]. [cited 2019 May 25]. Available from: https://www.powerthesaurus.org/

49. Princeton University. WordNet | A Lexical Database for English [Internet]. [cited 2019 May 23]. Available from: https://wordnet.princeton.edu/

50. What is Product Quality? Definition Meaning Importance [Internet]. [cited 2019 Apr 29]. Available from: https://kalyan-city.blogspot.com/2013/05/what-is-product-quality-definition.html

51. How to Improve Your Communication Skills by Understanding the Flow in Conversations | UniversalClass [Internet]. [cited 2019 Apr 30]. Available from: https://www.universalclass.com/articles/business/understanding-flow-in-conversation.htm

52. Shibl R, Lawley M, Debuse J. Factors influencing decision support system acceptance. Decis Support Syst. 2013 Jan 1;54(2):953–61.

1. Despite not being explicitly mentioned in the literature reviewed, in our evaluation projects, the assessment of knowledge and performance have emerged as potential determinants of behavioral change. [↑](#footnote-ref-2)
2. Despite not being explicitly mentioned in the literature reviewed, in our evaluation projects, the assessment of knowledge and performance have emerged as potential determinants of behavioral change. [↑](#footnote-ref-3)
